# Patterns and Correlates of Co-occurring Smoking, Alcohol Use, Gambling, and High Internet Use Among University Students: A Cross-Sectional Study in Türkiye

**DOI:** 10.1007/s10900-026-01563-0

**Published:** 2026-03-20

**Authors:** Ahmet Burak Avcu, Sena Nur Gündoğdu, Merve Sülü, Cengiz Çağdaş Kekilli, Gülseda Boz, Ayşe Baran, Metin Fikret Genç

**Affiliations:** 1 Department of Public Health, Inonu University School of Medicine, Malatya, Türkiye; 2 Department of Psychiatry, Nassau University Medical Center, East Meadow, NY USA

**Keywords:** Addictive behaviors, Alcohol, Gambling, Smoking, University students

## Abstract

University life may facilitate multiple addictive behaviors that tend to cluster rather than occur in isolation. The aim of this study was to estimate the prevalence and co-occurrence patterns of daily cigarette smoking, past-30-day alcohol use, gambling, and high daily internet use, and to evaluate their sociodemographic and behavioral associations among undergraduate students in Türkiye. This cross-sectional study was conducted at Inonu University (Malatya, Türkiye) between February–May 2024. Using stratified random sampling, n = 366 students were included. Outcomes were daily smoking, past-30-day alcohol use, past-year gambling, and high daily internet use (≥ 5 h/day). Analysis included UpSet framework for co-occurrence and logistic regression models. A Firth-penalized model assessed smoking factors. Prevalence was 39.1% for high internet use, 30.1% for daily smoking, 22.1% for gambling, and 7.7% for alcohol use. Overall, 59.0% reported at least one behavior, 27.6% reported ≥ 2, and 10.4% reported ≥ 3; the most common triple combination was high internet use, smoking, and gambling (6.3%). Male sex was associated with smoking (adjusted odds ratio [aOR] = 3.19) and gambling (aOR = 5.95). Smoking was independently associated with alcohol use (aOR = 5.34) and gambling (aOR = 2.25). High internet use was associated with highest family income (aOR = 2.81), alcohol (aOR = 2.54), and gambling (aOR = 2.73). Peer smoking showed an association with daily smoking (aOR = 17.25 for 2–3 friends vs. none). Risk behaviors are common and frequently cluster, particularly internet use, smoking, and gambling. These findings support integrated campus prevention and screening strategies that address multiple behaviors simultaneously rather than in isolation.

**Supplementary Information:**

The online version contains supplementary material available at 10.1007/s10900-026-01563-0.

## Introduction

The transition to university proceeds together with increased individual autonomy, changing friend groups, and new academic/social expectations. Under these conditions, health-related behaviors may become established and be carried into adulthood. In student populations smoking, alcohol use, gambling, and excessive daily internet use are important because each is associated with adverse health and functional outcomes and because these behaviors may be observed together rather than alone [1, 2].

Addictive behaviors can be considered persistent orientations toward rewarding activities despite negative outcomes and may encompass substance-related or behavioral domains [3, 4]. This framework is particularly meaningful for the university environment because shared determinants such as exposure to stress, campus life, and access to the digital world may simultaneously shape multiple behaviors and reveal patterns in which these behaviors are seen together [1].

Tobacco use remains to be one of the leading causes of preventable disease burden and deaths worldwide [5]. Alcohol use is associated with a wide range of harms, including many health problems and chronic disease burden [6]. Multinational studies conducted among university students show that tobacco use can frequently cluster with other health-risk behaviors, thereby supporting the value of a multidimensional approach in defining student risk profiles [2]. In gambling, sports betting and online betting have become increasingly accessible through digital platforms and have been associated with being observed together with substance-related risks in young people [7]. Similarly, although high daily internet use has now become a common practice among university students, heavy use has been reported to be associated with functional impairment and an increased likelihood of problematic internet use among students [8]. Meta-analysis evidence indicates that problematic internet use has been associated with other health-risk behaviors, including substance use and gambling-related outcomes; this suggests that it may point to overlapping vulnerabilities rather than independent exposures [9].

Clustering analyses in university samples show groups that have risk behaviors at the same time. These findings indicate that these multi behavior profiles tend to be associated with poorer mental health and broader functional impairment compared with single-behavior profiles [10–12]. There are important gaps in this field: many studies address these domains separately, evaluate only specific pairs, or use varying definitions, particularly for internet-related exposures. This reduces comparability and may render integrated profiles invisible [1, 8]. However, while it is likely that these behaviors cooccur and share common determinants, the number of studies that evaluate smoking, alcohol use, gambling participation, and high daily internet use in the same population, with consistent time windows, and within a single analytic framework is limited.

Accordingly, this study examines the prevalence and co-occurrence patterns of daily cigarette smoking, alcohol use in the past 30 days, any gambling in the past year, and high daily internet use among university students. By showing in which combinations these behaviors come together, the study aims to provide a basis for integrated prevention strategies that more accurately capture co-occurring risk profiles among students.

## Methods

### Study Design, Setting, and Participants

This cross-sectional study was conducted at Inonu University (Malatya, Türkiye) between February and May 2024 and is reported in accordance with the STROBE statement for cross-sectional studies. The source population comprised 23,446 registered undergraduate students in the 2023–2024 academic year.

The minimum sample size was n = 330, based on a reference prevalence of current smoking of 31.9% (95% confidence level; 5% margin of error). Anticipating nonresponse, we inflated the target by 20% (n = 396). Stratified random sampling was applied across 13 prespecified faculty strata. Within each stratum, enrollment lists were randomized using a computer-generated sequence, and students were approached sequentially until quotas were met.

**Fig. 1:**
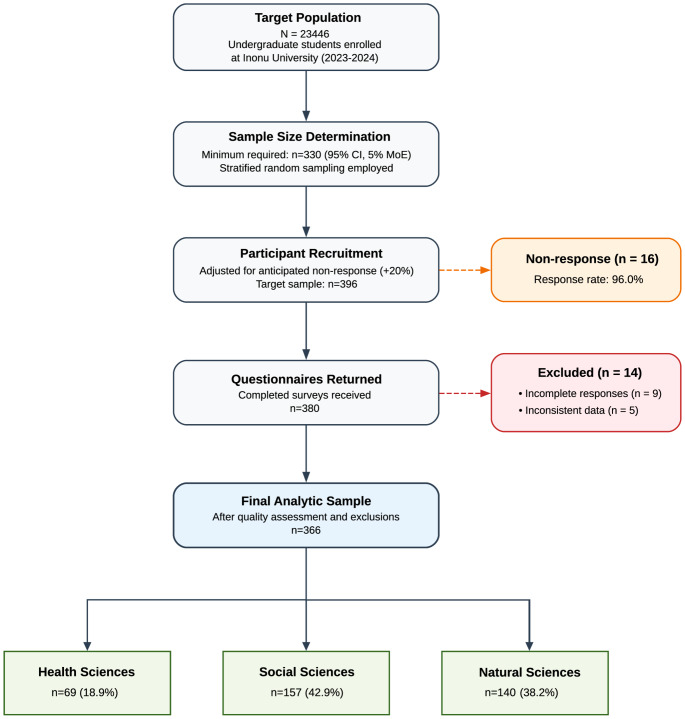
Participant flow diagram and final analytic sample (n = 366) from the source population of Inonu University undergraduates (N = 23,446), February to May 2024. Using stratified random sampling across 13 prespecified faculty/discipline strata, students were selected by simple random sampling within each stratum using a computer-generated random sequence, and n = 396 were invited between February and May 2024; n = 380 returned questionnaires (nonresponse n = 16; response rate 96.0%). After data quality checks, n = 14 questionnaires were excluded (incomplete responses n = 9; inconsistent/invalid per skip-logic checks n = 5), yielding a final analytic sample of n = 366

Of 396 invited students, 380 returned questionnaires (nonresponse n = 16; response rate 96.0%). After quality assessment, 14 questionnaires were excluded (incomplete n = 9; inconsistent with skip logic n = 5), yielding a final analytic sample of 366 participants (Fig. 1). Eligibility criteria were undergraduate enrollment, age ≥ 18 years, and written informed consent.

### Data Collection and Measurement

Data were collected using a structured questionnaire covering (1) sociodemographic characteristics; (2) tobacco, alcohol, illicit substance use, internet use, and gambling behaviors; and (3) potential correlates of these behaviors. Nicotine dependence among current smokers was assessed using the Heaviness of Smoking Index (HSI) [13]. To minimize social desirability bias, the questionnaire was self-administered on paper, completed privately, and returned in sealed opaque envelopes; no personal identifiers were recorded.

### Variables and Operational Definitions

#### Primary Outcomes

Current smoking was defined as daily cigarette smoking at the time of the survey (yes/no). Alcohol use in the past 30 days was defined as any alcohol consumption in the preceding 30 days (yes/no). Past-year gambling was defined as participation in ≥ 1 of the listed any gambling activities at least once in the past 12 months. High daily internet use was assessed using a past-week item (“In the last 7 days, what was your average total daily internet use?”) and dichotomized a priori as ≥ 5 versus < 5 h/day. Illicit drug use was summarized descriptively only due to very low prevalence.

#### Covariates

Sociodemographic covariates included gender, age group (18–20, 21–22, ≥ 23 years), academic domain, residence, monthly family income, and parental education. The 13 strata were consolidated into three prespecified academic domains: Health Sciences, Social Sciences, and Natural Sciences. Residence was categorized as dormitory/guesthouse, home/with family, or alone/with friends and dichotomized for regression as dormitory/guesthouse (reference) versus nondormitory. Monthly family income (TRY/month) was analyzed in categories aligned with the contemporaneous national monthly minimum wage at the time of data collection (17,002 TRY): ≤ 17,000 (≈ ≤ 1 × ; reference), 17,001–34,000 (≈1–2 ×), 34,001–51,000 (≈2–3 ×), and ≥ 51,001 (≈ ≥ 3 ×). Family smoking was coded as any smoking in the family (yes/no), and peer smoking was coded as the number of daily smokers among the three closest friends (0/1/2–3).

### Statistical Analysis

Analyses were conducted in R (version 4.5.2; R Foundation for Statistical Computing, Vienna, Austria). Descriptive statistics are presented as n (%) and as the mean (SD) or median (IQR), as appropriate. Separate logistic regression models were fitted for each primary outcome, reporting odds ratios (ORs) with 95% confidence intervals (CIs). Crude ORs were obtained from univariable models. Adjusted models were prespecified as Model A (sociodemographic covariates) and Model B (Model A plus the other primary behaviors, included according to the outcome, to assess behavioral clustering; interpreted associatively). For current smoking, robustness Model C used Firth’s penalized logistic regression (Model B plus family smoking and peer smoking). Multicollinearity was assessed using variance inflation factors. Discrimination was summarized using AUC and explained variation using Nagelkerke’s R^2^. Calibration was evaluated using diagnostic approaches acknowledging the limitations of the Hosmer‒Lemeshow test. Nested models were compared using likelihood ratio tests. All tests were two-tailed with p < 0.05.

### Ethics Approval and Consent

The study adhered to the Declaration of Helsinki and received approval from the Inonu University Ethics Committee (Approval No: 2024/5488; 03 May 2024). Written informed consent was obtained from all participants.

## Results

A total of 366 students were included in the analysis (56.8% women). The largest age group was 21–22 years (42.9%), followed by ≥ 23 years (33.1%) and 18–20 years (24.0%). Students were primarily enrolled in social sciences-related (42.9%) and natural sciences-related faculties (38.2%), with 18.9% in health-related faculties. Nearly half lived at home/with family (49.7%), 43.5% resided in dormitories/guesthouses, and 6.8% lived alone/with friends. Median monthly family income was 25.000 TRY (IQR: 17,000–40,000) and median individual income was 3.500 TRY (IQR: 2,000–5,000). Among participants’ three closest friends, 27.9% reported that all three smoked daily (Table 1).

**Table 1:** Sociodemographic and behavioral characteristics of the university student sample (N = 366)

Variable	n (%)
Age group (years)	
18–20	88 (24.0)
21–22	157 (42.9)
≥ 23	121 (33.1)
Gender	
Female	208 (56.8)
Male	158 (43.2)
Academic department	
Health-related faculties	69 (18.9)
Social sciences-related faculties	157 (42.9)
Natural sciences-related faculties	140 (38.2)
Residence	
Dormitory/guesthouse	159 (43.5)
Home/with family	182 (49.7)
Alone/with friends	25 (6.8)
Monthly family income (TRY)	25.000 (17.000–40.000)
Monthly individual income (TRY	3.500 (2.000–5.000)
Friends Who Smoke Daily (Among 3 Closest Friends)	
0	117 (32.0)
1	87 (23.8)
2	60 (16.4)
3	102 (27.9)
Tobacco and substance-related behaviors	
Ever tried cigarettes	183 (50.0)
Current tobacco smoking	110 (30.1)
Ever tried alcohol	100 (27.3)
Alcohol use in the past 30 days	28 (7.7)
Any gambling participation in past year	81 (22.1)
Ever use of illicit drugs	5 (1.4)
Daily internet use duration	
≤ 2 h	35 (9.6)
2–5 h	188 (51.3)
≥ 5 h	143 (39.1)

Data are presented as n (%) unless otherwise specified. Income is reported as the median (25th–75th percentile) in TRY. Current daily smoking was defined as daily cigarette use at the time of the survey

**Fig. 2:**
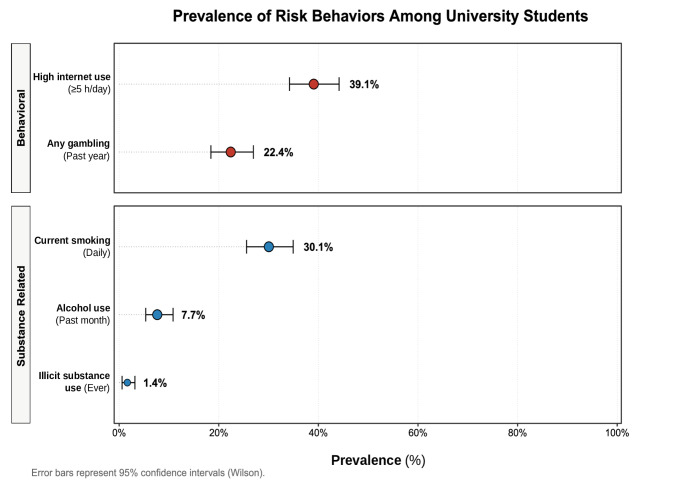
Prevalence of risk behaviors among undergraduate students at Inonu University, Türkiye (February–May 2024) (N = 366). Points indicate prevalence estimates, and horizontal bars denote 95% confidence intervals (Wilson). Prevalence is calculated using the full analytic sample (n = 366). High internet Use was defined as ≥ 5 h/day; Current Smoking as daily cigarette smoking; Any Gambling as participation in any gambling activity in the past year; Alcohol Use as any alcohol consumption in the past month; Illicit Substance Use as ever use. Measures are based on self-report

Regarding risk behaviors, 50.0% had ever tried cigarettes, and 30.1% reported current daily smoking. Ever alcohol use was reported by 27.3%, while past-30-day alcohol use was 7.7%. Any gambling in the past year was reported by 22.1%. Ever illicit drug use was rare (1.4%) and no current use was reported. High daily internet use (≥ 5 h/day) was the most prevalent behavior (39.1%) (Table 1; Fig. 2).

Among ever smokers (n = 183), the median age at smoking initiation was 17 years (16–19); among current smokers (n = 110), median consumption was 15 cigarettes/day (6–20). Over half of current smokers reported considering quitting (54.5%) and 61.1% reported a prior quit attempt (Supplementary Table S1). Among ever alcohol users (n = 100), the mean age at first alcohol use was 18 years (17–20), and use was predominantly infrequent (rare/special occasions 52.6%; tried once 24.2%); 28.0% of ever drinkers reported alcohol use in the past 30 days (Supplementary Table S2). By gambling activity (any frequency in the past year), participation was most common for sports betting (16.9%), followed by scratch cards/lottery (11.8%), online casino games (9.0%) (Supplementary Table S4). Nearly all participants used social media (97.8%), most commonly Instagram (92.5%) and YouTube (62.8%); 19.9% reported receiving warnings from family/friends due to excessive screen time, and 23.8% reported neglecting important tasks due to internet use (Supplementary Table S5).

High internet use (≥5 h/day) was the most common behavior, followed by current smoking and gambling; past-month alcohol use and ever illicit substance use were less frequently reported (Fig. 2).

**Fig. 3:**
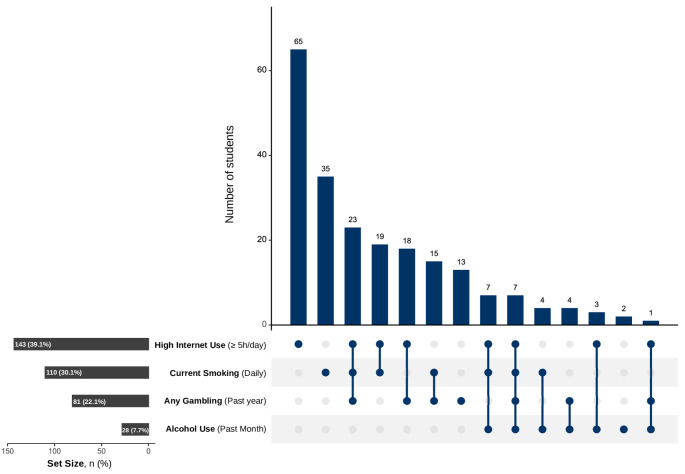
Co-occurrence patterns of High internet Use (≥ 5 h/day), Current Smoking (Daily), Any Gambling (Past year), and Alcohol Use (Past Month) (N = 366). The UpSet plot displays set sizes and intersection counts; connected dots indicate the behaviors included in each intersection

Co-occurrence patterns of High internet Use (≥ 5 h/day), Current Smoking (Daily), Any Gambling (Past year), and Alcohol Use (Past Month) are presented in Fig. 3. Overall, 59.0% (n=216) reported at least one of the four behaviors, whereas 41.0% (n=150) reported none. Exactly one behavior was observed in 31.4% (n=115); cooccurrence of ≥2 behaviors was observed in 27.6% (n=101), including 10.4% (n=38) reporting ≥3 behaviors. The most frequent multi-behavior intersection was High internet Use + Current Smoking + Any Gambling (6.3%, n=23), followed by High internet Use + Current Smoking (5.2%, n=19), High internet Use + Any Gambling (4.9%, n=18), and Current Smoking + Any Gambling (4.1%, n=15). The full four-behavior intersection was observed in 1.9% (n=7) (Fig. 3).

### Current Smoking

The smoking regression Table 2 presents the crude and adjusted associations with current smoking status (yes versus no). In univariable analyses, male students had significantly higher odds of smoking than female students (OR=4.47, 95% CI: 2.77–7.21; p<0.001). This association remained robust after adjustment for sociodemographic characteristics (Model A: aOR=3.89, 95% CI: 2.34–6.48; p<0.001) and after further adjustment for alcohol use in the past 30 days, gambling, and high daily internet use (Model B: aOR=3.19, 95% CI: 1.82–5.58; p<0.001).

**Table 2:** Factors associated with current smoking (yes vs no): crude and adjusted odds ratios from logistic regression (N = 366)

Variable	Crude OR (95% CI)	p value	Adjusted OR (95% CI), Model A	p value	Adjusted OR (95% CI), Model B	p value	Adjusted OR (95% CI), Model C	p value
Gender								
Male (ref Female)	4.47 (2.77–7.21)	< 0.001	3.89 (2.34–6.48)	< 0.001	3.19 (1.82–5.58)	< 0.001	2.14 (1.18–3.90)	0.012
Age group (years)								
21–22 (ref 18–20)	1.24 (0.69–2.25)	0.471	1.22 (0.64–2.32)	0.551	1.53 (0.77–3.04)	0.229	1.25 (0.60–2.65)	0.557
Age group (years)								
≥ 23 (ref 18–20)	1.59 (0.87–2.94)	0.134	1.44 (0.73–2.83)	0.291	1.61 (0.78–3.32)	0.200	1.42 (0.66–3.11)	0.370
Academic department								
Social sciences (ref Health-related)	1.44 (0.77–2.71)	0.254	1.37 (0.69–2.74)	0.369	1.55 (0.74–3.25)	0.243	1.27 (0.60–2.77)	0.539
Academic department								
Natural sciences (ref Health-related)	1.09 (0.57–2.10)	0.787	1.47 (0.72–3.03)	0.292	1.89 (0.88–4.07)	0.103	1.61 (0.73–3.69)	0.242
Residence								
Nondormitory (ref Dormitory)	1.16 (0.74–1.82)	0.522	1.00 (0.60–1.66)	0.989	0.92 (0.54–1.56)	0.754	0.85 (0.49–1.49)	0.574
Family income (TRY/month)								
17,001–34,000 (ref ≤ 17,000)	1.55 (0.84–2.88)	0.160	1.17 (0.60–2.26)	0.646	0.95 (0.48–1.89)	0.881	0.65 (0.30–1.38)	0.260
Family income (TRY/month)								
34,001–51,000 (ref ≤ 17,000)	1.67 (0.82–3.42)	0.157	1.42 (0.66–3.02)	0.366	1.18 (0.54–2.60)	0.682	0.89 (0.38–2.06)	0.779
Family income (TRY/month)								
≥ 51,001 (ref ≤ 17,000)	5.14 (2.24–11.83)	< 0.001	3.20 (1.31–7.78)	0.010	2.33 (0.91–5.94)	0.077	3.25 (1.18–9.37)	0.022
Alcohol (last 30 days)								
Yes (ref No)	4.81 (2.14–10.81)	< 0.001			5.34 (1.96–14.56)	0.001	4.54 (1.69–13.34)	0.002
Gambling (past year)								
Yes (ref No)	4.23 (2.52–7.10)	< 0.001			2.25 (1.22–4.15)	0.009	1.97 (1.03–3.78)	0.039
High daily internet use								
≥ 5 h/day (ref < 5)	2.01 (1.28–3.17)	0.003			1.33 (0.78–2.27)	0.303	1.13 (0.63–2.00)	0.684
Family smoker								
≥ 1 (ref None)	1.58 (0.99–2.52)	0.053					1.43 (0.82–2.53)	0.207
Close friends who smoke daily								
1 (ref 0)	7.13 (2.57–19.80)	< 0.001					6.13 (2.28–19.37)	< 0.001
Close friends who smoke daily								
2–3 (ref 0)	24.12 (9.35–62.21)	< 0.001					17.25 (6.99–51.78)	< 0.001

**Model fit. Model A:** AUC = 0.729 (0.672–0.786); AIC = 417.0; Nagelkerke R^2^ = 0.189; Omnibus likelihood-ratio (LR) χ^2^(10) = 52.45, p < 0.001; Hosmer‒Lemeshow χ^2^(5) = 1.61, p = 0.901; max adjusted GVIF = 1.04. **Model B:** AUC = 0.773 (0.721–0.826); AIC = 400.7; Nagelkerke R^2^ = 0.262; Omnibus LR χ^2^(13) = 74.76, p < 0.001; Hosmer‒Lemeshow χ^2^(5) = 13.70, p = 0.018; max adjusted GVIF = 1.12. Likelihood ratio test (Model B vs Model A): χ^2^(3) = 22.30, p < 0.001. **Model C (Firth):** AUC = 0.853 (0.813–0.892); Omnibus LR χ^2^(16) = 134.13, p < 0.001; Hosmer‒Lemeshow χ^2^(5) = 4.99, p = 0.417; max adjusted GVIF = 1.13. Likelihood ratio test (Model C vs Model B): χ^2^(3) = 59.37, p < 0.001. Note: For the penalized (Firth) model, AIC and pseudo-R^2^ were derived from the corresponding (nonpenalized) logistic model fitted on the same covariate set for comparability. Notes: Crude odds ratios (ORs) were obtained from univariable logistic regression models. Model A included gender, age group (18–20, 21–22, ≥ 23 years), academic department (health-related, social sciences-related, natural sciences-related), residence (Dorm vs Nondorm), and family income (TRY/month). Model B additionally included alcohol use in the last 30 days (yes/no), any gambling participation in past year (yes/no), and high daily internet use (≥ 5 h/day vs < 5 h/day). ORs are presented with 95% confidence intervals (CIs)

OR, odds ratio; CI, confidence interval

In the fully adjusted model, age group, academic department, residence, and most family-income categories were not independently associated with smoking. Students in the highest family-income group (≥51,001 TRY/month) showed increased odds of smoking in Model A (aOR=3.20, 95% CI: 1.31–7.78; p=0.010), but the association attenuated and did not reach conventional statistical significance after inclusion of behavioral covariates (Model B: aOR=2.33, 95% CI: 0.91–5.94; p=0.077). In Model B, alcohol use in the last 30 days (aOR=5.34, 95% CI: 1.96–14.56; p=0.001) and gambling (aOR=2.25, 95% CI: 1.22–4.15; p=0.009) were independently associated with higher odds of current smoking, whereas high daily internet use (≥5 h/day) was not (aOR=1.33, 95% CI: 0.78–2.27; p=0.303) (Table 2).

**Fig. 4:**
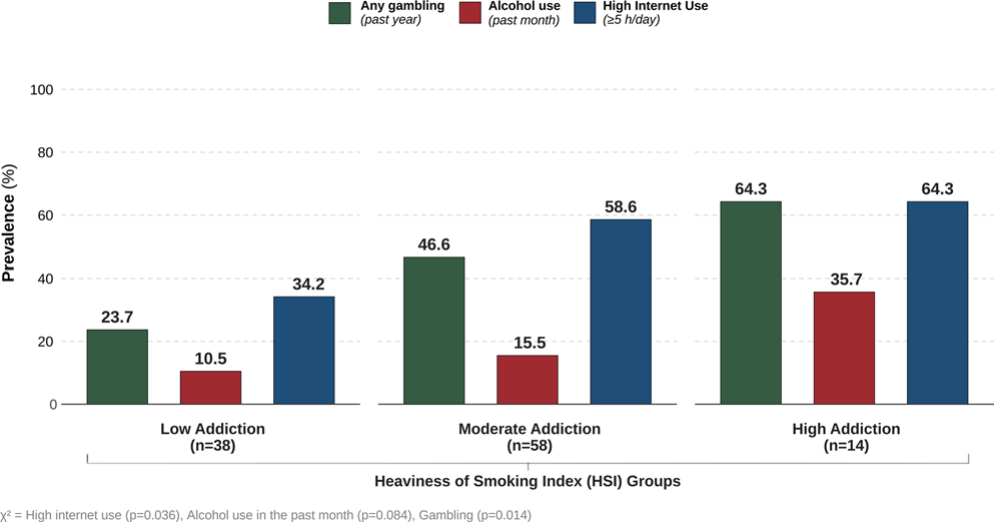
Prevalence of gambling, alcohol use, and high internet use across nicotine dependence severity (HSI groups) among current smokers (N = 110). Values represent within-group prevalence (%) among current smokers in each HSI category. Gambling corresponds to Any Gambling (Past year); Alcohol Use corresponds to Alcohol Use (Past Month); High internet Use was defined as ≥ 5 h/day. p values indicate differences across HSI categories (χ^2^ test); p < 0.05 was considered statistically significant. HSI, Heaviness of Smoking Index

Among current smokers, the prevalence of cooccurring risk behaviors increased monotonically with higher nicotine dependence severity (Fig. 4). Gambling participation increased across HSI categories (p = 0.014), and high internet use (≥ 5 h/day) showed a similar increasing pattern (p = 0.036). Alcohol use in the past month also increased with higher HSI levels, although the trend did not reach conventional statistical significance (p = 0.084) (Fig. 4).

### Alcohol Use in the Past 30 Days

Alcohol use varied by age and academic department. Compared with students aged 18–20 years, those aged 21–22 years had lower odds of alcohol use after adjustment (Model A: aOR = 0.27, 95% CI: 0.08–0.88; p = 0.030), with a similar association after further behavioral adjustment (Model B: aOR = 0.22, 95% CI: 0.06–0.80; p = 0.022). Academic department was also associated with alcohol use: relative to health-related departments, students in social sciences had lower odds (Model B: aOR = 0.26, 95% CI: 0.09–0.74; p = 0.012), and those in natural sciences had substantially lower odds (Model B: aOR = 0.12, 95% CI: 0.03–0.45; p = 0.001).

Family income demonstrated a dose‒response pattern, with the highest income category (≥ 51,001 TRY/month) associated with greater odds of alcohol use in both models (Model A: aOR = 9.26, 95% CI: 1.61–53.31; p = 0.013; Model B: aOR = 6.38, 95% CI: 1.03–39.60; p = 0.047). In Model B, current smoking was associated with alcohol use (aOR = 4.77, 95% CI: 1.73–13.17; p = 0.003). High daily internet use showed borderline evidence of association (aOR = 2.63, 95% CI: 0.99–6.99; p = 0.053), whereas gambling was not independently associated (aOR = 1.88, 95% CI: 0.65–5.46; p = 0.244). Gender and residence were not significant predictors in the fully adjusted model (Table 3).

**Table 3:** Factors associated with alcohol use in the past 30 days (yes vs no): crude and adjusted odds ratios from logistic regression (Models A and B) (N = 366)

Variable	Crude OR (95% CI)	p value	Adjusted OR (95% CI), Model A	p value	Adjusted OR (95% CI), Model B	p value
Gender						
Male (ref Female)	1.57 (0.73–3.41)	0.251	1.14 (0.47–2.74)	0.776	0.40 (0.13–1.21)	0.103
Age group (years)						
21–22 (ref 18–20)	0.33 (0.10–1.04)	0.058	0.27 (0.08–0.88)	0.030	0.22 (0.06–0.80)	0.022
Age group (years)						
≥ 23 (ref 18–20)	1.42 (0.57–3.50)	0.453	0.84 (0.30–2.31)	0.731	0.88 (0.30–2.63)	0.822
Academic department						
Social sciences (ref Health-related)	0.36 (0.15–0.86)	0.021	0.36 (0.14–0.94)	0.037	0.26 (0.09–0.74)	0.012
Academic department						
Natural sciences (ref Health-related)	0.18 (0.06–0.52)	0.002	0.19 (0.06–0.61)	0.005	0.12 (0.03–0.45)	0.001
Residence						
Nondormitory (ref Dormitory)	2.45 (1.02–5.92)	0.046	1.64 (0.64–4.22)	0.301	1.51 (0.56–4.12)	0.419
Family income (TRY/month)						
17,001–34,000 (ref ≤ 17,000)	4.83 (1.05–22.14)	0.043	4.08 (0.85–19.65)	0.080	4.03 (0.79–20.58)	0.094
Family income (TRY/month)						
34,001–51,000 (ref ≤ 17,000)	2.24 (0.36–13.79)	0.385	2.52 (0.39–16.05)	0.329	2.17 (0.32–14.77)	0.428
Family income (TRY/month)						
≥ 51,001 (ref ≤ 17,000)	9.41 (1.80–49.21)	0.008	9.26 (1.61–53.31)	0.013	6.38 (1.03–39.60)	0.047
Current smoking (daily)						
Yes (ref No)	4.81 (2.14–10.81)	< 0.001			4.77 (1.73–13.17)	0.003
Gambling (past year)						
Yes (ref No)	2.92 (1.32–6.47)	0.008			1.88 (0.65–5.46)	0.244
High daily internet use						
≥ 5 h/day (ref < 5)	3.07 (1.37–6.85)	0.006			2.63 (0.99–6.99)	0.053

**Model fit. Model A:** AUC = 0.802 (0.721–0.882); AIC = 189.1; Nagelkerke R^2^ = 0.193; Omnibus LR χ^2^(10) = 30.66, p < 0.001; Hosmer‒Lemeshow χ^2^(5) = 2.59, p = 0.763; max adjusted GVIF = 1.08. **Model B:** AUC = 0.865 (95% CI: 0.791–0.939); AIC = 172.3; Nagelkerke R^2^ = 0.326; Omnibus LR χ^2^(13) = 53.49, p < 0.001; Hosmer‒Lemeshow χ^2^(5) = 6.78, p = 0.237; max adjusted GVIF = 1.35. Likelihood ratio test (Model B vs Model A): χ^2^(3) = 22.83, p < 0.001. Notes: Crude odds ratios (ORs) were obtained from univariable logistic regression models. Model A included gender, age group (18–20, 21–22, ≥ 23 years), academic department (health-related, social sciences, natural sciences), residence (Dormitory vs Nondormitory), and family income (TRY/month). Model B additionally included current smoking (yes/no), gambling (yes/no), and high daily internet use (≥ 5 h/day vs < 5 h/day). ORs are presented with 95% confidence intervals (CIs)

OR, odds ratio; CI, confidence interval

### Gambling

Factors associated with gambling (past year; yes vs no) are summarized in the gambling regression table. Male gender emerged as the strongest correlate: male students had statistically significant higher odds of gambling than female students in crude analysis (OR = 8.39, 95% CI: 4.60–15.29; p < 0.001), and the association persisted after adjustment (Model A: aOR = 7.43, 95% CI: 3.95–13.97; p < 0.001; Model B: aOR = 5.95, 95% CI: 3.06–11.58; p < 0.001). Age group, academic department, residence, and income were not independently associated with gambling in Model B.

In the behavioral model, current smoking (aOR = 2.18, 95% CI: 1.18–4.01; p = 0.012) and high daily internet use (≥ 5 h/day; aOR = 2.67, 95% CI: 1.47–4.85; p = 0.001) were independently associated with higher odds of gambling, whereas alcohol use in the last 30 days was not (aOR = 1.74, 95% CI: 0.64–4.72; p = 0.276) (Table 4).

**Table 4:** Factors associated with gambling (past year) (yes vs no): crude and adjusted odds ratios from logistic regression (Models A and B) (N = 366)

Variable	Crude OR (95% CI)	p value	Adjusted OR (95% CI), Model A	p value	Adjusted OR (95% CI), Model B	p value
Gender						
Male (ref Female)	8.39 (4.60–15.29)	< 0.001	7.43 (3.95–13.97)	< 0.001	5.95 (3.06–11.58)	< 0.001
Age group (years)						
21–22 (ref 18–20)	0.89 (0.47–1.70)	0.731	0.77 (0.38–1.60)	0.489	0.88 (0.40–1.92)	0.750
Age group (years)						
≥ 23 (ref 18–20)	1.25 (0.65–2.40)	0.501	1.08 (0.51–2.28)	0.841	1.28 (0.57–2.86)	0.550
Academic department						
Social sciences (ref Health-related)	1.53 (0.77–3.03)	0.222	1.61 (0.74–3.51)	0.226	1.62 (0.71–3.69)	0.249
Academic department						
Natural sciences (ref Health-related)	0.77 (0.37–1.61)	0.492	1.08 (0.47–2.48)	0.864	1.12 (0.46–2.71)	0.806
Residence						
Nondormitory (ref Dormitory)	1.50 (0.90–2.51)	0.117	1.22 (0.68–2.19)	0.495	1.18 (0.64–2.16)	0.596
Family income (TRY/month)						
17,001–34,000 (ref ≤ 17,000)	2.79 (1.33–5.85)	0.007	1.87 (0.83–4.20)	0.133	1.74 (0.74–4.05)	0.201
Family income (TRY/month)						
34,001–51,000 (ref ≤ 17,000)	2.70 (1.17–6.24)	0.020	2.40 (0.96–6.02)	0.061	2.04 (0.78–5.29)	0.144
Family income (TRY/month)						
≥ 51,001 (ref ≤ 17,000)	3.89 (1.52–9.95)	0.005	1.86 (0.67–5.17)	0.234	1.00 (0.33–3.02)	0.994
Current smoking (daily)						
Yes (ref No)	4.23 (2.52–7.10)	< 0.001			2.18 (1.18–4.01)	0.012
Alcohol (last 30 days)						
Yes (ref No)	2.92 (1.32–6.47)	0.008			1.74 (0.64–4.72)	0.276
High daily internet use						
≥ 5 h/day (ref < 5)	3.11 (1.87–5.18)	< 0.001			2.67 (1.47–4.85)	0.001

**Model fit. Model A:** AUC = 0.796 (0.741–0.851); AIC = 331.7; Nagelkerke R^2^ = 0.292; Omnibus LR χ^2^(10) = 77.23, p < 0.001; Hosmer‒Lemeshow χ^2^(5) = 2.64, p = 0.755; max adjusted GVIF = 1.04. **Model B:** AUC = 0.840 (95% CI: 0.792–0.887); AIC = 312.8; Nagelkerke R^2^ = 0.373; Omnibus LR χ^2^(13) = 102.12, p < 0.001; Hosmer‒Lemeshow χ^2^(5) = 2.30, p = 0.806; max adjusted GVIF = 1.07. Likelihood ratio test (Model B vs Model A): χ^2^(3) = 24.89, p < 0.001. Notes: Crude odds ratios (ORs) were obtained from univariable logistic regression models. **Model A** included gender, age group (18–20, 21–22, ≥ 23 years), academic department (health-related, social sciences, natural sciences), residence (Dormitory vs Nondormitory), and family income (TRY/month). **Model B** additionally included current smoking (yes/no), alcohol use in the past 30 days (yes/no), and high daily internet use (≥ 5 h/day vs < 5 h/day). ORs are presented with 95% confidence intervals (CIs)

OR, odds ratio; CI, confidence interval

### High Daily Internet Use

In the sociodemographic model, male students had higher odds of high internet use (Model A: aOR = 1.61, 95% CI: 1.01–2.57; p = 0.043), but this association attenuated and was not significant after inclusion of behavioral covariates (Model B: aOR = 1.10, 95% CI: 0.65–1.86; p = 0.717). Students aged ≥ 23 years had lower odds of high internet use than those aged 18–20 years in both models (Model A: aOR = 0.50, 95% CI: 0.27–0.91; p = 0.025; Model B: aOR = 0.46, 95% CI: 0.25–0.87; p = 0.016).

Higher family income was associated with greater odds of high internet use, with the highest income group (≥ 51,001 TRY/month) remaining significant in both models (Model A: aOR = 3.41, 95% CI: 1.45–8.03; p = 0.005; Model B: aOR = 2.81, 95% CI: 1.15–6.86; p = 0.023). In Model B, alcohol use in the last 30 days (aOR = 2.54, 95% CI: 1.02–6.33; p = 0.046) and any gambling in past year (aOR = 2.73, 95% CI: 1.51–4.92; p < 0.001) were independently associated with high internet use, while current smoking was not (aOR = 1.32, 95% CI: 0.78–2.25; p = 0.303) (Table 5).

**Table 5:** Factors associated with high daily internet use (≥ 5 h/day vs < 5 h/day): crude and adjusted odds ratios from logistic regression (Models A and B) (N = 366)

Variable	Crude OR (95% CI)	p value	Adjusted OR (95% CI), Model A	p value	Adjusted OR (95% CI), Model B	p value
Gender						
Male (ref Female)	1.69 (1.11–2.59)	0.015	1.61 (1.01–2.57)	0.043	1.10 (0.65–1.86)	0.717
Age group (years)						
21–22 (ref 18–20)	0.89 (0.52–1.50)	0.658	0.84 (0.49–1.45)	0.532	0.90 (0.51–1.58)	0.711
Age group (years)						
≥ 23 (ref 18–20)	0.60 (0.34–1.05)	0.075	0.50 (0.27–0.91)	0.025	0.46 (0.25–0.87)	0.016
Academic department						
Social sciences (ref Health-related)	1.24 (0.69–2.23)	0.465	1.03 (0.56–1.89)	0.932	1.05 (0.55–2.00)	0.891
Academic department						
Natural sciences (ref Health-related)	1.07 (0.59–1.95)	0.819	1.00 (0.53–1.87)	0.998	1.11 (0.57–2.16)	0.754
Residence						
Nondormitory (ref Dormitory)	1.16 (0.76–1.77)	0.500	1.19 (0.76–1.88)	0.441	1.12 (0.70–1.78)	0.638
Family income (TRY/month)						
17,001–34,000 (ref ≤ 17,000)	1.12 (0.64–1.98)	0.683	0.98 (0.54–1.76)	0.944	0.84 (0.45–1.54)	0.564
Family income (TRY/month)						
34,001–51,000 (ref ≤ 17,000)	1.20 (0.62–2.33)	0.591	1.10 (0.56–2.18)	0.779	0.93 (0.46–1.89)	0.850
Family income (TRY/month)						
≥ 51,001 (ref ≤ 17,000)	3.83 (1.69–8.71)	0.001	3.41 (1.45–8.03)	0.005	2.81 (1.15–6.86)	0.023
Current smoking (daily)						
Yes (ref No)	2.01 (1.28–3.17)	0.003			1.32 (0.78–2.25)	0.303
Alcohol (last 30 days)						
Yes (ref No)	3.07 (1.37–6.85)	0.006			2.54 (1.02–6.33)	0.046
Gambling (past year)						
Yes (ref No)	3.11 (1.87–5.18)	< 0.001			2.73 (1.51–4.92)	< 0.001

**Model fit. Model A:** AUC = 0.614 (0.553–0.674); AIC = 489.6; Nagelkerke R^2^ = 0.080; Omnibus LR χ^2^(10) = 22.19, p = 0.014; Hosmer‒Lemeshow χ^2^(5) = 2.12, p = 0.833; max adjusted GVIF = 1.07. **Model B:** AUC = 0.675 (0.615–0.734); AIC = 471.8; Nagelkerke R^2^ = 0.160; Omnibus LR χ^2^(13) = 45.91, p < 0.001; Hosmer‒Lemeshow χ^2^(5) = 4.58, p = 0.469; max adjusted GVIF = 1.18. Likelihood ratio test (Model B vs Model A): χ^2^(3) = 23.71, p < 0.001. Notes: Crude odds ratios (ORs) were obtained from univariable logistic regression models. **Model A** included gender, age group (18–20, 21–22, ≥ 23 years), academic department (health-related, social sciences, natural sciences), residence (Dormitory vs Nondormitory), and family income (TRY/month). **Model B** additionally included current smoking (yes/no), alcohol use in the past 30 days (yes/no), and gambling (past year) (yes/no). ORs are presented with 95% confidence intervals (CIs)

OR, odds ratio; CI, confidence interval

## Discussion

In this cross-sectional study of 366 university students, smoking, alcohol use, gambling, and high daily internet use frequently co-occurred rather than occurring in isolation. High daily internet use (≥ 5 h/day) was most prevalent (39.1%), followed by daily smoking (30.1%) and past-year gambling (22.1%), while past-30-day alcohol use was lower (7.7%). Overall, 59.0% reported at least one of these behaviors and 27.6% reported two or more, with intersections most often involving high internet use combined with smoking and gambling. Male sex and peer smoking were associated with daily smoking, smoking was independently associated with alcohol use and gambling, and high internet use was independently associated with alcohol use and gambling. These results may suggest that students’ addictive behavior risk profiles are better understood as behavioral clusters.

### Daily Cigarette Smoking

Daily cigarette smoking was common in our university sample (30.1% overall; 47.5% in men and 16.8% in women). Half of students reported having ever tried cigarettes. When compared against national surveillance, this level is notable: GATS Türkiye 2016 reported 29.5% daily smoking overall (41.8% men; 17.4% women), indicating that our student estimate is close to adult population levels rather than a student/youth profile [14]. A more recent national indicator similarly places daily tobacco use among adults aged ≥ 15 years in 2022 at 28.3% (41.3% men; 15.5% women) [15].

In Iran, a systematic review/meta-analysis of university studies estimated 16% overall smoking (26% men; 7% women) [16], and a large national student sample reported 6.0% [17]. In contrast, some university settings report levels comparable to or higher than ours (e.g., 34.1% past-30-day smoking in Bosnia and Herzegovina) [18], while a regional systematic review in Arab countries documented very high current smoking in some settings, with a consistently higher prevalence among men [19]. Taken together, these comparisons underscore substantial cross-country variability and suggest that our estimate is best interpreted within Türkiye’s high-background tobacco context [14, 15].

Smoking initiation patterns in our sample show that smoking often starts before a student reaches university. Among current smokers, 23.3% initiated smoking before age 15, and 50.0% initiated smoking between 15 and 18 years (median initiation age 17). Initiation patterns also match the numbers from GATS Türkiye 2016 which reports an initiation age of 17, 15.0 percent starting before age 15 and 57.5 percent starting before age 18 [14].

Current smoking was also appeared a part of risky behaviors set instead of happening alone. In our data, smoking was concentrated among men, showed a strong gradient with peer smoking and co-occurred with past-month alcohol use and past-year gambling. This clustering is consistent with university evidence that smoking commonly aligns with risky alcohol patterns [20, 21], while the smoking-gambling relationship may be more context-dependent after wider adjustment in some settings [22].

### Alcohol Use

In our sample, past-30-day alcohol use was 7.7%, making it the least prevalent of the four risk behaviors assessed. This estimate is lower than many recent university-based reports internationally, such as Myanmar (20.3% past 30 days) [23] and Ethiopia (26.2% one-month pooled prevalence across student groups) [24]. Even when studies report “excessive” rather than any-use measures, prevalence can remain substantially higher (e.g., Brazil: 18.7% excessive alcohol consumption) [25]. Within Türkiye, our past-month estimate is also below the prevalence of hazardous alcohol consumption (13.5%) reported in a large student sample while noting that hazardous drinking and any past-month use reflect different constructs and are not directly interchangeable [26].

Beyond overall prevalence, alcohol use in our data showed meaningful subgroup patterning. Alcohol use was less common among students aged 21–22 than 18–20 and was lowest in social sciences and natural sciences compared with health-related faculties. While multicountry syntheses often report higher alcohol use among men and with increasing age, predictors vary by setting and by how alcohol use is operationalized (e.g., any use vs hazardous use) [27]. Our faculty gradient is also consistent with evidence that the field of study can shape alcohol risk in university settings [25]. Higher family income emerged as the clearest socioeconomic correlate in our sample, supporting the interpretation that greater disposable resources and access may translate into a higher likelihood of alcohol use in student populations [27].

Finally, our findings reinforce alcohol’s co-occurrence with other risk behaviors in university settings. Alcohol use was more common among current daily smokers, aligning with evidence that smoking and alcohol frequently cluster among students and young adults across settings [23, 28]. In contrast, alcohol use was not independently patterned by gambling after accounting for covariates—consistent with the possibility that alcohol-gambling links are context- and outcome-dependent (e.g., stronger for gambling problems than for any participation in some surveys) [22]. We also observed suggestive evidence that high daily internet use (≥ 5 h/day) may accompany alcohol use; this direction is coherent with meta-analytic findings linking problematic internet use with increased risk of substance use outcomes [9]. Collectively, even where past-month alcohol use is relatively uncommon, it may concentrate within multiple addictive behavior profiles (especially alongside smoking), suggesting that integrated screening and combined risk-reduction approaches might be more efficient than single-behavior strategies [23, 26].

### Any Gambling

In our sample, any gambling in the past year was 22.1%, and the pattern was highly concentrated rather than diffuse. Sports betting showed a pronounced male pattern (37.3% in men vs 1.4% in women) and increased steeply across income categories (from 4.3% in ≤ 17,000 TRY to 37.1% in ≥ 51,001 TRY). The prevalence was highest among students living alone/with friends (32.0%), whereas age group and academic department did not show meaningful differences.

When comparing findings across studies, it is essential to distinguish participation from disorder. Meta-analytic evidence focused on pathological gambling among college students typically indicates a prevalence of 6.13%, which is not directly comparable to our outcome of any past-year gambling participation [29]. Prevalence estimates vary substantially across studies, partly due to heterogeneity in operational definitions (e.g., any participation vs. problem/disorder; lifetime vs. past-year) and the gambling modalities captured [29, 30]. In contexts where sports betting predominates, even participation-based measures can yield high engagement; for example, a Nigerian university sample reported 30.3% past-year sports betting, and 64.2% of past-year bettors reported negative academic impacts [31]. Similarly, among Nigerian medical and dental students, a multi-institutional survey reported an overall gambling prevalence of 40.19%, although 10.42% screened positive for a possible gambling disorder [32]. Broader participation-based definitions may therefore yield substantially higher prevalence estimates; for example, in a Spanish sample of 735 young students in Madrid, 42.6% reported having engaged in sports betting at least once [33]. A post-COVID survey of adolescents and young adults (including university-age groups) in Spain reported high lifetime exposure, with a large share of individuals who had ever gambled also participating in the prior year [34].

Gambling also appeared embedded within a co-occurring risk profile in our study. Participation was more common among current daily smokers and among students with high daily internet use (≥ 5 h/day), consistent with the notion that digital environments can amplify access and reinforcement opportunities and that multiple risk behaviors can cluster in adolescents and young adults [9]. Reviews focused on problematic online gambling further emphasize its overlap with other online/addictive behaviors and the measurement challenges that complicate comparisons across studies [30]. In contrast, we did not observe an independent pattern by past-month alcohol use after accounting for covariates. This differs from some higher-education surveys in which risky alcohol use shows association with gambling problems [22] and may reflect differences in outcome definition (any gambling vs problem gambling) and the relatively low alcohol prevalence in our sample. Overall, the findings may support prevention strategies that prioritize male students and sports betting and that integrate tobacco and digital-environment risk rather than treating gambling as an isolated behavior.

### High Daily Internet Use (≥ 5 h/day)

High daily internet use was common in our sample, with 39.1% of students reporting ≥ 5 h/day. Men exceeded this threshold more often than women (46.2% vs 33.8%). In the cross-study context, this estimate is broadly consistent with time-based findings from other university settings, where substantial proportions of students report spending > 4–7 h/day online depending on the population and cutoff used [35, 36]. Notably, much of the literature operationalizes exposure as problematic internet use (PIU) rather than time alone; however, syntheses consistently identify time spent online as a central, dose-related marker associated with PIU and related impairment [8]. Meta-analysis evidence from African high school and university populations indicates that exceeding 4 h per day is significantly associated with an increased risk of internet addiction symptoms, reinforcing the notion that prolonged daily exposure may signify increased susceptibility among certain students [37].

Our multivariable patterning suggests that the observed sex difference in heavy use may be partly attributable to cooccurring risk behaviors rather than sex per se: the male excess attenuated after accounting for alcohol use and gambling. We also observed a clear socioeconomic gradient in daily hours, with the highest-income group most likely to report ≥ 5 h/day. In contrast, older students (≥ 23 years) were less likely to report high daily internet use, consistent with the possibility that role transitions and reduced discretionary time shift online behavior away from prolonged daily exposure in later university years.

High daily internet use also clustered with other addictive behaviors. After behavioral covariates were included, alcohol use and gambling remained independently associated with high internet use, whereas smoking was not. This pattern is coherent with broader evidence that PIU co-occurs with alcohol-related risk behaviors in university-age populations [38] and with meta-analytic findings linking PIU/internet addiction constructs to a wider cluster of health-risk behaviors, including substance use outcomes [9]. In addition, evidence from higher-education settings indicates that digital behaviors and gambling-related harms can overlap, highlighting the importance of considering “screen time” within a broader digital-risk context rather than as an isolated exposure [22]. Taken together, our findings support integrated prevention approaches that prioritize students presenting with concurrent heavy internet use alongside alcohol use and gambling, which may share common drivers such as stress coping, reward sensitivity, and digital availability.

### Limitations

This study has several limitations. First, its cross-sectional design precludes causal inference and prevents establishing temporal ordering between behaviors; therefore, findings should be interpreted as concurrent co-occurrence patterns rather than causal effects. Second, outcomes were based on self-reports and may be affected by recall error and social desirability bias particularly for alcohol use and gambling in potentially stigmatizing contexts so some underreporting and misclassification may persist despite anonymous, sealed-envelope procedures. Third, we used pragmatic, time-windowed definitions rather than clinical constructs: high internet use (≥ 5 h/day) captured exposure time but not problematic use or functional impairment, and gambling reflected any past-year participation without distinguishing recreational from problem/disordered gambling, which may limit comparability with studies using validated severity or diagnostic tools. Fourth, the study was conducted at a single university in a specific regional setting, which may constrain generalizability; although stratified random sampling and a high response rate support internal validity, selection bias remains possible if nonresponders differed systematically. Finally, we did not measure key psychosocial determinants (e.g., distress, impulsivity, academic stress, family functioning), so residual confounding is possible, and observed clustering may partly reflect unmeasured shared drivers; nonetheless, the study adds value by assessing four commonly cooccurring risk behaviors within the same population using consistent time windows and an integrated framework that emphasizes multibehavior intersections rather than isolated outcomes.

## Conclusions

In this study, risk behaviors were common and frequently co-occurred rather than appearing in isolation. Overall, 59.0% of students reported at least one of the four behaviors, and more than a quarter reported two or more behaviors. The most frequent co-occurring behaviors involved high internet use combined with smoking and/or gambling, indicating that student risk profiles may be better characterized as clusters rather than single exposures. Across models, male gender showed association with both smoking and gambling, while alcohol use and gambling were consistently associated with smoking, and gambling and alcohol use were associated with high internet use. These findings may support the need for integrated prevention and screening strategies on campuses that address multiple behaviors simultaneously, rather than siloed, single-behavior programs. Practically, screening for these behaviors in student health services, targeted interventions for higher-risk groups, and combined counseling approaches may better reflect clustering patterns.

To better establish temporal precedence, future studies should use multi-center longitudinal designs and validated measures that integrate both mental health outcomes and psychosocial determinants. Such work would help determine whether the observed intersections represent shared vulnerabilities, contextual influences, or pathways linking behaviors over time.

## Supplementary Information

Below is the link to the electronic supplementary material.

Supplementary file1 (DOCX 27 KB)

## Data Availability

The dataset generated and analyzed during the current study is stored in CSV format. De-identified data can be obtained from the corresponding author upon reasonable request, subject to ethical approval and institutional guidelines.
