# Supplementary material for: Patterns and Correlates of Co-occurring Smoking, Alcohol Use, Gambling, and High Internet Use Among University Students: A Cross-Sectional Study in Türkiye
Source: J Community Health. 2026 Mar 20;51(3):479–93. doi: 10.1007/s10900-026-01563-0 (PMC13249695; doi:10.1007/s10900-026-01563-0)
Supplement: Supplementary file 1 — Supplementary file1 (DOCX 27 KB) [file 10900_2026_1563_MOESM1_ESM.docx]

Supplementary File 1. Additional descriptive characteristics of risk behaviors.

**Table S1.** Tobacco use characteristics (N=366)

| **Characteristic** | **n (%)** | **Median (IQR)** |
| --- | --- | --- |
| **Ever smoked cigarettes (n=366)** | 183 (50.0) |  |
| **Age at smoking initiation, years (ever smokers, n=183)** |  | 17 (16-19) |
| **Current daily smoking (n=366)** | 110 (30.1) |  |
| **Cigarettes per day (current smokers, n=110)** |  | 15 (6-20) |
| **Primary reason for smoking initiation (current smokers, n=110)** |  |  |
| Friends/family influence | 29 (26.4) |  |
| Curiosity | 24 (21.8) |  |
| Imitation / wanting to fit in | 11 (10.0) |  |
| Major life event (e.g., death/illness) | 10 (9.1) |  |
| Loneliness | 9 (8.2) |  |
| University-related stress | 8 (7.3) |  |
| Family problems | 4 (3.6) |  |
| Other | 15 (13.6) |  |
| **When smoking increases most (current smokers, n=110)** |  |  |
| When with friends | 22 (20.0) |  |
| When responsibilities increase | 18 (16.4) |  |
| Exam periods | 16 (14.5) |  |
| Financial difficulties | 2 (1.8) |  |
| Other | 11 (10.0) |  |
| No change | 41 (37.3) |  |
| **Considering quitting (current smokers, n=110)** |  |  |
| Yes | 60 (54.5) |  |
| No | 50 (45.5) |  |
| **Ever attempted to quit (current smokers with data, n=108)** |  |  |
| Yes | 66 (61.1) |  |
| No | 42 (38.9) |  |
| **Reasons for wanting to quit* (among quit attempters, n=66)** |  |  |
| Harmful to health | 63 (95.5) |  |
| Economic reasons | 21 (31.8) |  |
| Family or social pressure | 3 (4.5) |  |
| Religious reasons | 3 (4.5) |  |
| Other | 5 (7.6) |  |

**Note.** Data are presented as n (%) unless otherwise indicated. Age at smoking initiation and cigarettes per day are reported as median (IQR; 25^th^-75^th^ percentile). *Multiple-response item; percentages are calculated using the stated denominator and may sum to >100%.

**Table S2.** Alcohol use characteristics (N=366)

| **Characteristic** | **n (%)** | **Median (IQR)** |
| --- | --- | --- |
| **Ever used alcohol (n=366)** | 100 (27.3) |  |
| **Age at first alcohol use, years (among ever alcohol users, n=100)** |  | 18.0 (17-20) |
| **Reason for trying/using alcohol (respondents with data, n=96)** |  |  |
| Curiosity | 52 (54.1) |  |
| Enjoyment | 20 (20.8) |  |
| Friends’ influence | 16 (16.7) |  |
| To relieve distress | 4 (4.2) |  |
| Used in the family | 2 (2.1) |  |
| Other | 2 (2.1) |  |
| **Alcohol use frequency (respondents with data, n=99)** |  |  |
| Daily | 3 (3.0) |  |
| 1-2 days/week | 3 (3.0) |  |
| 1-2 days/month | 17 (17.2) |  |
| Rarely / special occasions | 52 (52.6) |  |
| Tried only once | 24 (24.2) |  |
| **Any alcohol use in the past 30 days (n=366)** |  |  |
| Yes | 28 (7.7) |  |
| No | 338 (92.3) |  |
| **Attempted to quit alcohol use (respondents with data, n=88)** |  |  |
| Yes | 26 (29.5) |  |
| No | 62 (70.5) |  |

Note. Data are presented as n (%) unless otherwise indicated. Age at first alcohol use is reported as median (IQR; 25^th^-75^th^ percentile). Past-30-day alcohol use was defined as any alcohol consumption in the preceding 30 days. Percentages are calculated using the stated denominator for each item; denominators may vary due to missing responses.

**Table S3.** Substance use characteristics (N=366)

| **Characteristic** | **n (%)** |
| --- | --- |
| **Ever use of an illicit/addictive substance (n=366)** | 5 (1.4) |
| **Current use of an illicit/addictive substance (n=366)** | 0 (0.0) |

Note. Data are presented as n (%).

**Table S4.** Gambling behaviors (N=366)

| **Characteristic** | **n (%)** |
| --- | --- |
| **Horse racing (n=366)** |  |
| Never | 358 (97.8) |
| <1 time/week | 4 (1.1) |
| ≥1 time/week | 4 (1.1) |
| **Card games for money (n=366)** |  |
| Never | 333 (91.0) |
| <1 time/week | 25 (6.8) |
| ≥1 time/week | 8 (2.2) |
| **Sports betting / match predictions (n=366)** |  |
| Never | 304 (83.1) |
| <1 time/week | 41 (11.2) |
| ≥1 time/week | 21 (5.7) |
| **Scratch cards / lottery (n=366)** |  |
| Never | 323 (88.2) |
| <1 time/week | 34 (9.3) |
| ≥1 time/week | 9 (2.5) |
| **Online casino games (n=366)** |  |
| Never | 333 (91.0) |
| <1 time/week | 22 (6.0) |
| ≥1 time/week | 11 (3.0) |
| **Returning to gamble to recover losses (respondents with data, n=244)** |  |
| Never | 202 (82.8) |
| Sometimes (<50% of the time) | 27 (11.1) |
| Often (most times) | 7 (2.9) |
| Always | 8 (3.3) |

Note. Data are presented as n (%). Gambling frequency categories refer to the past 12 months.

**Table S5.** Internet/technology use characteristics (N=366)

| **Characteristic** | **n (%)** |
| --- | --- |
| **Daily internet use (hours/day; n=366)** |  |
| <1 hour | 7 (1.9) |
| 1-2 hours | 28 (7.7) |
| 2-3 hours | 58 (15.8) |
| 3-4 hours | 130 (35.5) |
| ≥5 hours | 143 (39.1) |
| **Uses ≥1 social media platforms (n=366)** | 358 (97.8) |
| **Social media platforms used (multiple responses allowed) (n=358)** |  |
| Instagram | 331 (92.5) |
| YouTube | 225 (62.8) |
| X (Twitter) | 181 (50.6) |
| Facebook | 79 (22.1) |
| **Received warnings from family/friends due to excessive screen time (n=366)** |  |
| Yes | 73 (19.9) |
| No | 162 (44.3) |
| Sometimes | 131 (35.8) |
| **Neglected important tasks due to internet use (n=366)** |  |
| Yes | 87 (23.8) |
| No | 160 (43.7) |
| Sometimes | 119 (32.5) |

**Note.** Data are presented as n (%). Daily internet use categories reflect the past-week average of total daily internet use (hours/day).Percentages for social media platforms are calculated among social media users (n=358). Multiple responses allowed; totals may exceed 100%. Percentages are calculated using the stated denominator for each item; denominators may vary due to missing responses.
